# Increased Expression of *SETD7* Promotes Cell Proliferation by Regulating Cell Cycle and Indicates Poor Prognosis in Hepatocellular Carcinoma

**DOI:** 10.1371/journal.pone.0154939

**Published:** 2016-05-16

**Authors:** Yuanyuan Chen, Shengsheng Yang, Jiewei Hu, Chaoqin Yu, Miaoxia He, Zailong Cai

**Affiliations:** 1 Department of Biochemistry and Molecular Biology, Second Military Medical University, Shanghai, 200433, China; 2 Department of Pathology, Changhai Hospital, Second Military Medical University, Shanghai, 200433, China; 3 Department of Gynecology of Traditional Chinese Medicine, Changhai Hospital, Second Military Medical University, Shanghai, 200433, China; The University of Hong Kong, HONG KONG

## Abstract

**Purpose:**

To investigate the role of SET domain containing 7 (SETD7) in hepatocellular carcinoma (HCC) and determine whether SETD7 can be used as a predictor of overall survival in HCC patients.

**Methods:**

mRNAs and proteins of *SETD7* and related genes in HCC tumor samples and paired adjacent non-tumorous liver tissues (ANLTs) (n = 20) or culture cells were determined by quantitative real-time PCR and Western blot. Cell proliferation and apoptosis with SETD7 knockdown SMMC-7721 cells or SETD7 overexpressed HepG2 cells were analyzed by CCK8 assay or flow cytometry. Gene expression alterations in SETD7 knockdown of SMMC-7721 cells were determined by digital gene expression (DGE) profiling. Defined data on patients (n = 225) with HCC were retrieved for the further study. Tissue microarrays (TMAs) were performed using paraffin tissues with tumor and ANLTs. SETD7 and related proteins were determined by TMAs immunohistochemistry. Statistical analyses were conducted to associate SETD7 expression with tumor features and patient outcomes, as well as related proteins expression.

**Results:**

SETD7 expression was significantly higher in HCC tumor tissues than in ANLTs. SETD7 overexpression in vitro can promote HepG2 cell proliferation, whereas SETD7 knockdown can inhibit SMMC-7721 cell proliferation by regulating the cell cycle. SETD7 expression was significantly correlated with five genes expression. Increased SETD7 is associated with metastasis, recurrence, large tumor size, and poor tumor differentiation, and indicates poor prognosis in HCC patients.

**Conclusions:**

SETD7 plays a critical role in HCC, and its immunohistochemistry signature provides potential clinical significance for personalized prediction of HCC prognosis.

## Introduction

Hepatocellular carcinoma (HCC) is one of the most common malignant tumors worldwide. In China, HCC is the third leading cause of morbidity and the second leading cause of mortality among malignancies, with a total mortality rate of 26.26 per 100,000[1]. The cause of HCC is multifaceted, that is, complex genome and epigenetic alterations, including changes in histone modification, DNA methylation, abnormal microRNA expression, and epigenetic regulation of the altered gene expression, which are all correlated with the development and progression of HCC [2]. Histone 3 lysine 4 (H3K4) specific histone methyltransferases (HMTs) catalyze H3K4 methylation, which is generally associated with gene activation. Dysregulated expression of H3K4 HMTs and their genetic mutations lead to malignant progression [3].

As a methyltransferase for H3K4, *SETD7* (also known as SET7, SET9, or SET7/9) belongs to the SET domain-containing proteins, which can change the chromatin state by influencing the binding abilities of the cofactor to the histone via direct histone methylation, which is associated with demethylation of H3K4 (H3K4me2) and promotes downstream gene expression [4–11]. Moreover, SETD7 potentially regulates proteins, modulates transcription factor activity, and activates promoters of methylation-dependent co-recruitment by mediated methylation of non-histone proteins [12]. The presence of multifarious substrates suggests the manifold biological functions of SETD7. Reports indicate that SETD7 plays an important role in inflammation, metabolism-associated diseases, viral infection, and oncogenesis. In type 2 diabetes mellitus, hyperglycemia induces upregulation of *SETD7*, leading to monomethylation of H3K4 in the NF-κB p65 promoter and contributing to vascular dysfunction [13]. SETD7 suppresses the IFN receptors and facilitates hepatitis C virus (HCV) replication. *SETD7* is highly expressed in Huh7.5.1 cells infected with HCV, as well as in the plasma, peripheral blood mononuclear cells, and hepatic tissues of patients infected with HCV [14]. *SETD7* with frameshift mutation in castration-resistant prostate cancer [15] is the downstream target of miR-153; overexpression of miR-153 also promotes degradation of SETD7 and then suppresses ovarian cancer cell proliferation and invasion[16]. However, the functions and mechanisms of SETD7 in HCC remain poorly understood.

Thus, in the present study, we examined the expression of *SETD7* in HCC tumor tissues and ANLTs. In vitro, *SETD7* knockdown, *SETD7* overexpression, and DGE analysis were performed to explore the functions and mechanisms of SETD7 in regulating cell growth. Immunohistochemistry (IHC) was performed in tissue microarrays (TMAs) to estimate the expression of *SETD7* associated with HCC occurrence and progression, as well as its relevance to the prognosis.

## Materials and Methods

### Patients and samples

20 pairs of HCC tumor tissues and adjacent non-tumorous liver tissues (ANLTs) were surgically collected at the general surgery department, and 225 pairs of paraffin-embedded tissues, including HCC tumor tissues and ANLTs, were obtained from the pathology department of Changhai Hospital between 2009 and 2013. The patients had been diagnosed with HCC according to the WHO Classification of Tumor of the Digestive System. Clinical data, including patient characteristics, clinical presentation, tumor differentiation, sites of lesion, laboratory findings, objective response, and survival were collected from the hospital information system. The summary of clinicopatholgic characteristics are in S1 Table. All patients provided informed written consent for sample collection and permission to use for research purposes. The protocol for all experiments was approved by Ethics Committee of the Second Military Medical University.

### Cell culture

The cell lines SMMC-7721, HepG2, QGY-7703, Bel-7404, HCC-0010 and HL-7702 were purchased from the Type Culture Collection of the Chinese Academy of Sciences (Shanghai, China). Cells were cultured in Dulbecco’s Modified Eagle’s medium (DMEM) (Hyclone, USA) with 10% fetal bovine serum (FBS), at 37°C in a humidified incubator containing 5% CO_2_.

### RNA extraction, reverse transcription and qRT-PCR

The total RNA was isolated from tissues or cells using the TRIzol reagent (Invitrogen). The cDNA was synthesized following the manufacturer’s instructions (ThermoFisher). qRT-PCR of *SETD7*, zinc finger and BTB domain containing 20 (*ZBTB20*), cyclin-dependengt kinase inhibitor 2D (*CDKN2D*) were performed using FastStart Universal SYBR Green Master (Rox) (Roche). qRT-PCR assay were performed using an ABI quantitative PCR model 7300 (Applied Biosystems). Primers for *SETD7*, *ZBTB20*, *CDKN2D*, *GAPDH* were designed as follows S2 Table. *GAPDH* was used as internal reference. The qRT-PCR was performed in triplicate and included no-template controls. Relative expression was calculated with the comparative CT method.

### Western blot and IHC assay

Cells or tissues were lysed and western blot was performed as previously described [17], S3 Table lists the antibodies used in this research.

TMAs were constructed from the paraffin embedded tissues that surgically resected HCC tissues and ANTLs, then cut into 4-μm-thick serial sections. IHC was performed as protocols provided by the manufacturers. Scores were independently assessed by two pathologists blinded to clinical data. Staining intensity: 0 (negative), 1 (weak), 2 (moderate), 3 (strong); percent positivity: 0 (0%-5%), 1 (5%-25%), 2 (25%-50%), 3 (50%-100%). The final expression score was counted with the staining intensity score plus percent positivity score, and scores 0–2 defined as negative expression, scores 3–6 defined as positive expression [18].

### *SETD7* silencing sequences and overexpression plasmid

3 interfering RNAs (siRNA) targeting human *SETD7* and the scrambled siRNA were designed and synthesized by GenePharma (Shanghai, China) (S4 Table). The *SETD7* overexpression or control plasmid was constructed by Genechem as S1 Fig.

### Cell proliferation, cell cycle and apoptosis analysis

For cell proliferation, SMMC-7721 cells were plated at a concentration of 5×10^3^ cells each well in 96-well plates that media containing 10% FBS, after incubation for 24 h, the cells were transfected with si-*SETD7*-2 or the scrambled RNA at a final concentration of 100 nM using FuGENE HD Transfection Reagent (Roche, Basel, Switzerland), according to the manufacturer’s protocol. Meanwhile, HepG2 cells were transfected with pGV141-*SETD7* or pGV141 plasmid in similar way (0.2 μg plasmid/ per well). Cell proliferation assay was performed by Cell Counting Kit-8 (CCK-8) solution (Obio technology, Shanghai, China) according to the manufacturer’s protocol. For cell cycle and apoptosis analysis, cells were plated at a concentration of 5×10^5^ cells each well in 6-well plates, and transfected in the same way. After incubation for 36 h, cells were trypsinized, collected after washing twice with phosphate-buffered saline, fixed in 75% cold ethanol, then follows cell cycle and apoptosis analysis protocol (BD) and performed using a FACS cytometer (FACS, CA, USA).These experiments were performed in triplicate.

### Digital gene expression profiling (DGE) and bioinformatics

After SMMC-7721 cells was transfected with si-*SETD7*-2 or the scrambled RNA for 24 h, the cells were harvested in TRIzol reagent (*SETD7* knockdown efficiency were confirmed) then sent to DGE analysis. Sequencing was performed at RiboBio Co., Guangzhou through the illumineHiSeq2500. Data was aligned to the Ensembl transcript annotations using bowtie and RSEM as described previously [19]. All other bioinformatics analysis was performed using glbase [20].

### Statistical analysis

Results were analyzed with SPSS 21.0 statistical software. Student’s t test was used to evaluate the quantitative variables. Pearson test or correction for continuity chi square test was used to evaluate the correlation between *SETD7* expression and clinicopathologic parameters. Kaplan-Meier analysis was used to estimate the survival probability. Log-rank test was used to estimate the comparison of survival curves between groups. Univariate and multivariate Cox regression analysis was used to determine contribution of *SETD7* expression to the survivals. Significance was defined as p< 0.05.

## Results

### Expression of *SETD7* in HCC tumor tissues, paired ANLTs and cell lines

**Fig 1:**
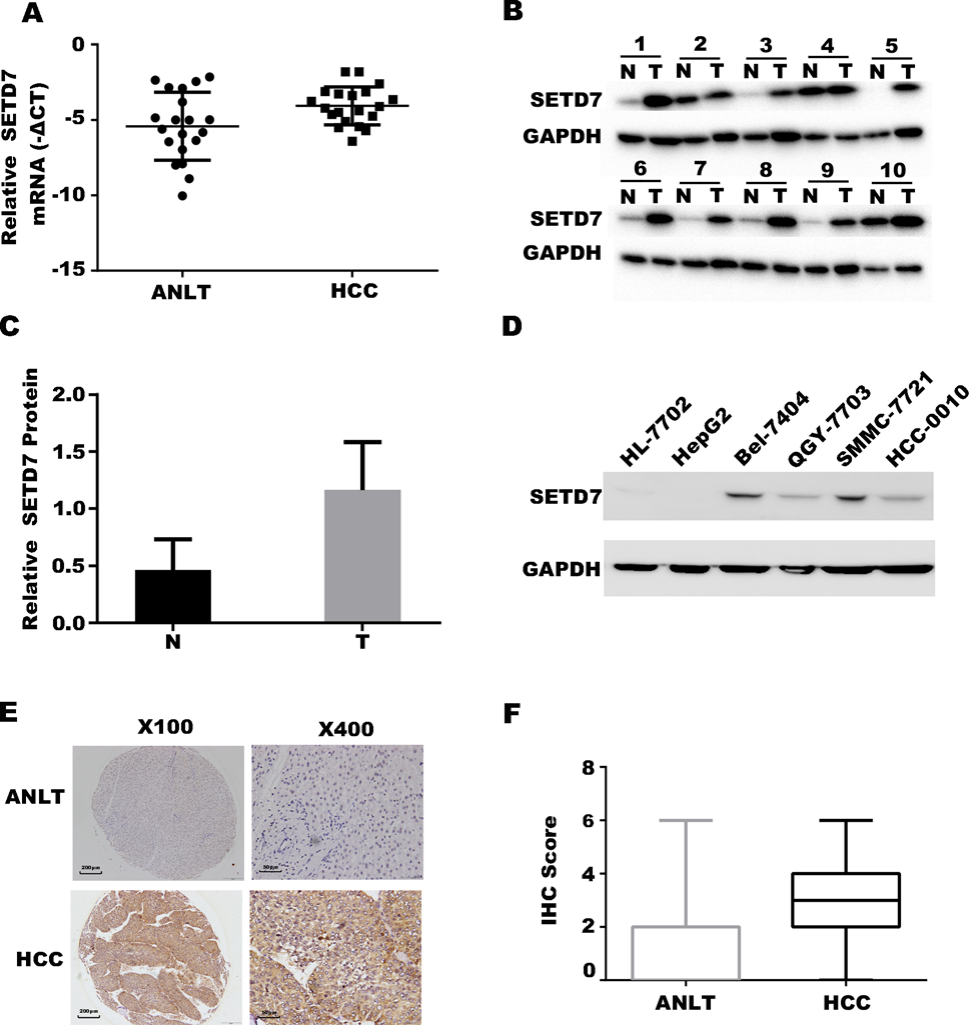
Expression of *SETD7* in HCC tumor tissues, paired ANLTs and cell lines. (A) qRT-PCR analysis was performed to analyze *SETD7* expression in 20 pairs of HCC tumor tissues and ANLTs, the data shown are the mean of –ΔCT, and the expression of *SETD7* in HCC is significantly higher than that in ANLTs (P<0.05); (B) Western blot assay was performed to detect *SETD7* expression in 20 pairs of HCC tumor tissues and ANLTs; (C) Gray value of Western blot result (P<0.05); (D) Western blot assay was performed to analyze *SETD7* expression in normal hepatocyte cell line (HL-7702) and liver cancer cell lines (HepG2, SMMC-7721, QGY-7703, HCC-0010, and Bel-7404); (E). Typical IHC staining of SETD7 in HCC tumor tissues and ANLTs (with figures on the left magnified 100× and figures on the right magnified 400×); (F) Box plots indicate the IHC scores of SETD7 in HCC tumor tissues and ANLTs [mean, 3.204 (SD, 0.092) vs 1.427 (SD, 0.083), P<0.01].

*SETD7* exhibited a markedly higher expression in HCC tumor tissues than in ANLTs, as determined by qRT-PCR (Fig 1A). This finding was verified by Western blot analysis (Fig 1B); the gray value of the Western blot result was calculated (Fig 1C). SMMC-7721, QGY-7703, Bel-7404, HCC-0010, HepG2, and HL-7702 cells were detected by Western blot analysis to determine the SETD7 expression in different liver cancer cell lines and the normal liver cell line. The results exhibit as following (in descending order of their SETD7 levels): SMMC-7721, Bel-7404, QGY-7703, and HCC-0010. Almost negative results were obtained in HepG2 and HL-7702 (Fig 1D). IHC assay revealed that SETD7 is significantly higher in HCC tumor tissues than in ANLTs (Fig 1E). The IHC scores of SETD7 in HCC tumor tissues and ANLTs are indicated in the box plots (Fig 1F).

### *SETD7* expression changes liver cancer cell proliferation by regulating the cell cycle

**Fig 2:**
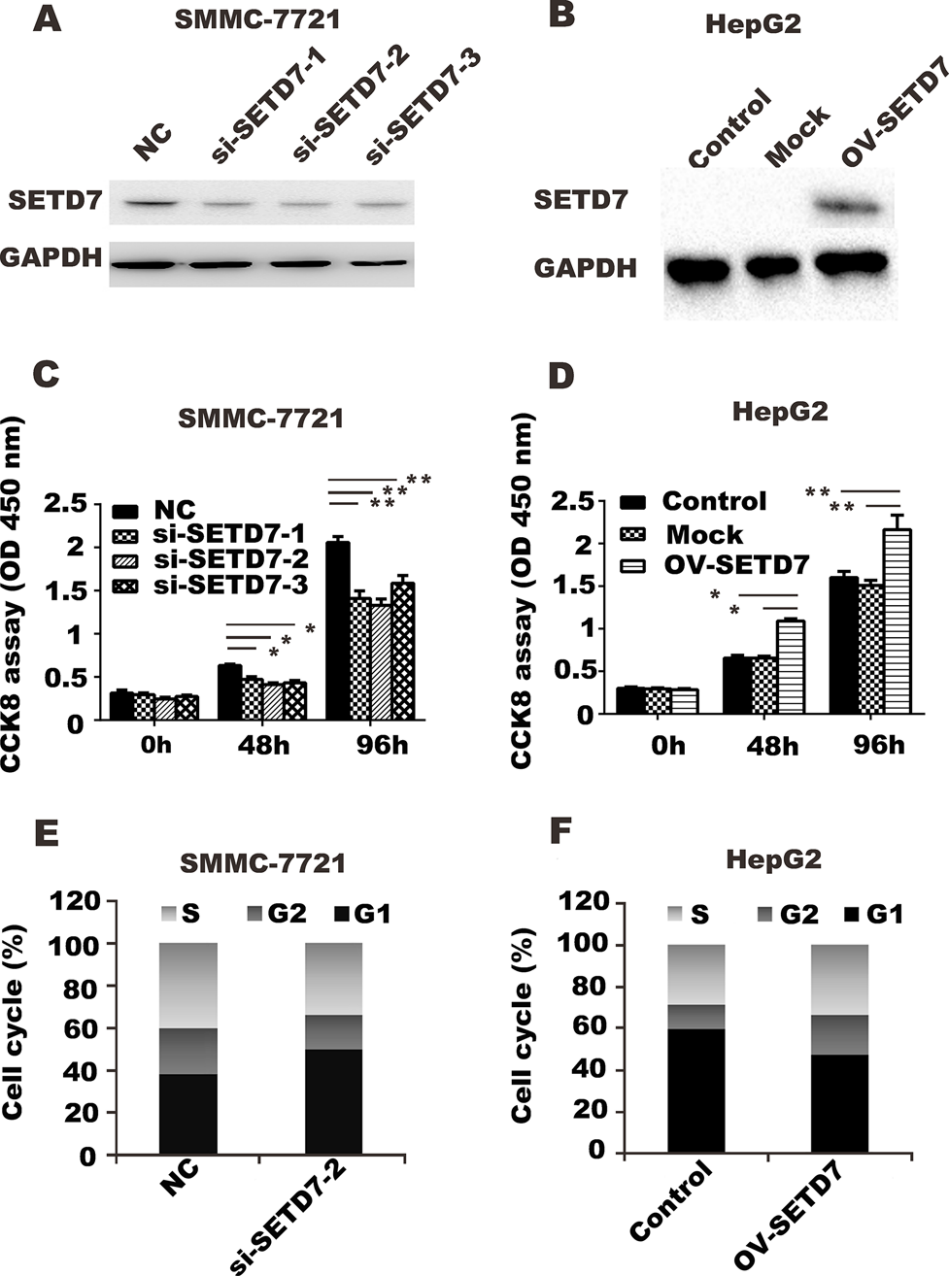
*SETD7* expression changes liver cancer cell proliferation by regulating the cell cycle. (A) Western blot analysis was performed to detect the interference efficiency of siRNA after transfected with si-*SETD7*-1, si-*SETD7*-2, si-*SETD7*-3, or scramble RNA (NC) in SMMC-7721. (B) Western blot analysis was performed to detect *SETD7* expression in HepG2 transfected with pGV141-*SETD7* (OV-*SETD7*) or pGV141 plasmid (Control). (C, D) CCK8 array was used to assess proliferation in *SETD7* knockdown in SMMC-7721 cells or *SETD7* overexpression in HepG2 cells. *, P<0.05; **, P<0.01 by student’s t test. (E, F) Cell cycle was assessed in *SETD7* knockdown in SMMC-7721 cells or *SETD7* overexpression in HepG2 cells by flow cytometry assay.

Considering the higher expression of *SETD7* in HCC tumor tissues than in ANLTs, we determined whether SETD7 would affect cell function. Three interference sequences S1, S2, and S3 were designed to knock down *SETD7* in SMMC-7721. *SETD7* overexpression plasmid was constructed and transfected into HepG2 cells. Interfering efficiency and overexpression capacity were detected by Western blot analysis after transfection for 24 h; specifically, three interference sequences obtained almost same knockdown efficiency (around 40–50%) (Fig 2A and 2B). CCK8 assay showed that *SETD7* knockdown inhibited SMMC-7721 cell growth, whereas *SETD7* overexpression increased HepG2 cell growth, compared with the control group (Fig 2C and 2D).

To explore the approach of *SETD7* in regulating cell proliferation, apoptosis was detected in SMMC-7721 and HepG2. Apoptosis rates were not altered significantly in *SETD7* knockdown SMMC-7721 cells or in *SETD7* overexpressed HepG2 cells, compared with the control group (data not shown). Cell-cycle assay revealed that cells in the G1 phase were significantly decreased in *SETD7* knockdown SMMC-7721 cells, compared with the control (Fig 2E); this result showed an opposite effect on HepG2 cells with *SETD7* overexpressed (Fig 2F).

### DGE analysis of *SETD7* knockdown in SMMC-7721 cells

**Fig 3:**
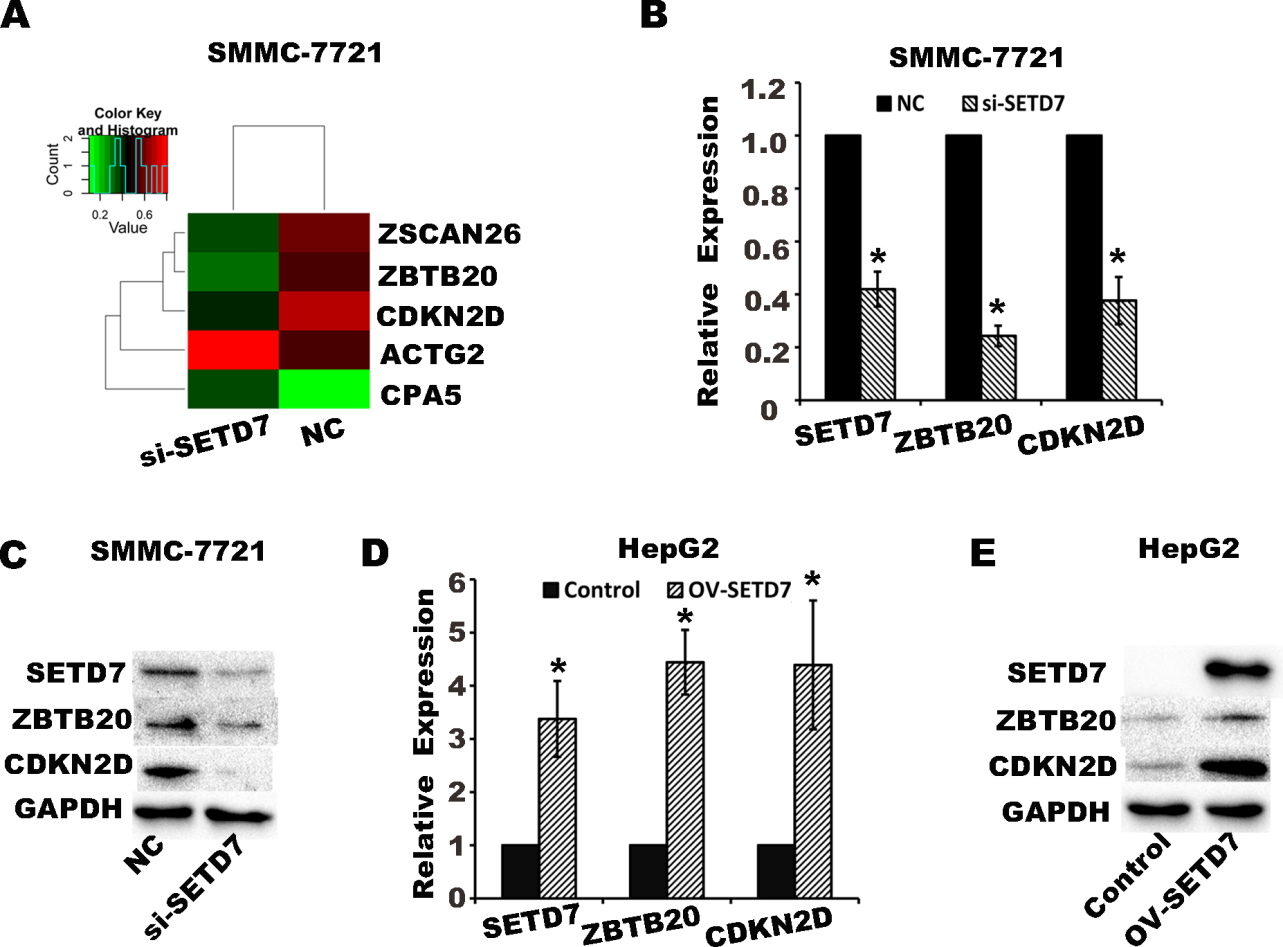
SETD7 regulates the expression of ZBTB20 and CDKN2D. (A) Differential gene expression profile was explored and analyzed after *SETD7* knockdown in SMMC-7721, as shown in Heatmap (q-value<0.05). (B, C) qRT-PCR and western blot analysis indicate that after *SETD7* knockdown in SMMC-7721, the expression levels of ZBTB20 and CDKN2D were significantly downregulated. *P< 0.05 by Student’s t-test. (D, E) qRT-PCR and western blot analysis indicated the ZBTB20 and CDKN2D were significantly upregulated in *SETD7* overexpression in HepG2 cells. *P< 0.05 by Student’s t-test.

To elucidate the mechanism underlying *SETD7* regulation of cell proliferation, DGE analysis was performed in *SETD7* knockdown SMMC-7721 cells by transfecting the S2 interference sequence. The results showed that ZBTB20, zinc finger and SCAN domain-containing protein 26 (ZSCAN26), and CDKN2D were significantly downregulated, whereas actin, gamma 2, smooth muscle, enteric (ACTG2) and carboxypeptidase A5 (CPA5) were significantly upregulated after *SETD7* knockdown (Fig 3A). For ZBTB20 and CDKN2D correlate with cell cycle, the mRNA and protein levels of ZBTB20 and CDKN2D were subsequently verified by qRT-PCR and Western blot analysis in *SETD7* knockdown SMMC-7721 cells and in *SETD7* overexpressed HepG2 cells (Fig 3B–3E).

### SETD7 correlated with H3K4me2, ZBTB20, and CDKN2D via TMA IHC analysis

**Fig 4:**
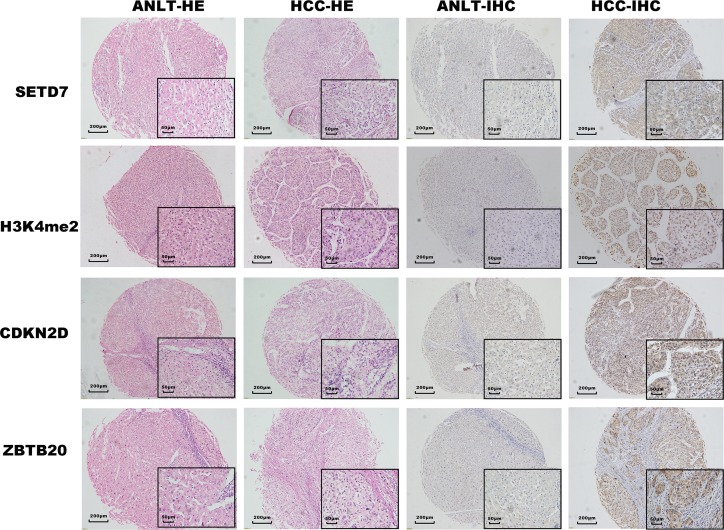
SETD7, H3K4me2, ZBTB20, and CDKN2D level in HCC was determined by TMA IHC. The two rows on the left indicate HE staining for HCC tumor tissues and paired ANLTs, whereas the two rows on the right indicate IHC staining for SETD7, H3K4me2, ZBTB20, and CDKN2D protein in HCC tumor tissues and paired ANLTs (*n* = 225). (Original magnified 100×; Inserted figures magnified 400×).

To verify whether SETD7 is more highly expressed in HCC tumor tissues than in ANLTs and that SETD7 correlates with ZBTB20 and CDKN2D in vitro, TMA IHC staining for SETD7, H3K4me2, ZBTB20, and CDKN2D was performed on the 225 pairs of HCC tumor tissues and ANLTs (Fig 4). SETD7, CDKN2D and ZBTB20 are widely expressed in the nuclei and cytoplasms of HCC specimens. H3K4me2 immunoreactivity was detected in the nuclei. As expected, SETD7, H3K4me2, CDKN2D, and ZBTB20 are significantly higher in HCC tumor tissues than in ANLTs (Fig 4). The expression of SETD7 is closely related to the expression of ZBTB20 (P = 0.002), H3K4me2 (P = 0.04), and CDKN2D (P = 0.004) in HCC (Table 1).

**Table 1:** Correlation between the SETD7 and H3K4me2, ZBTB20, CDKN2D expression in HCC.

	SETD7 expression			
Gene products	Low n(%)	High n(%)	χ²	*p* value
**H3K4me2**			**4.78**	**0.04**
**Low**	**28 (12.4%)**	**36 (16.0%)**		
**High**	**46 (20.4%)**	**115 (51.1%)**		
**ZBTB20**			**10.366**	**0.002**
**Low**	**29 (12.9%)**	**29 (12.9%)**		
**High**	**45 (20.0%)**	**122 (54.2%)**		
**CDKN2D**			**9.311**	**0.004**
**Low**	**23 (10.2%)**	**21 (9.3%)**		
**High**	**51 (22.7%)**	**130 (57.8%)**		

### Relationship between SETD7 expression and clinicopathologic parameters

To further investigate the clinical significance of SETD7 expression in the development and progression of HCC, primary HCC samples were divided into two groups, low expression of SETD7 (IHC score: 0–2, n = 74) and high expression of SETD7 (IHC score: 3–6, n = 151). Analysis indicated high expression of SETD7 in HCC tumor tissues is significantly correlated with several crucial clinicopathologic parameters, such as metastasis (P = 0.009), recurrence (P = 0.012), poor tumor differentiation (P = 0.047), and large tumor size (P = 0.001), which are associated with the increased risk of early recurrence and reduced survival in HCC patients (Table 2) [21].

**Table 2:** Correlation between the SETD7 and clinicopatologic variables in HCC.

	SETD7 expression			
Clinical characters	Low n (%)	High n (%)	χ²	*p* value
**Age (y)**			**0.107**	**0.744**
**≤55**	**37 (16.4%)**	**79 (35.1%)**		
**>50**	**37 (16.4%)**	**72 (32.0%)**		
**Gender**			**0.005**	**0.946**
**Male**	**60 (26.7%)**	**123 (54.7%)**		
**Female**	**14 (6.2%)**	**28 (12.4%)**		
**Liver cirrhosis**			**0.476**	**0.49**
**No**	**16 (7.1%)**	**39 (17.3%)**		
**Yes**	**58 (25.8%)**	**112 (49.8%)**		
**AFP (ng/ml)**			**0.717**	**0.397**
**≤200**	**47 (20.9%)**	**87 (38.7%)**		
**>200**	**27 (12.0%)**	**64 (28.4%)**		
**Tumor differentiation**			**4.175**	**0.047**
**I-II**	**46 (20.4%)**	**72 (32.0%)**		
**III-IV**	**28 (12.4%)**	**79 (35.1%)**		
**Tumor size (cm)**			**10.762**	**0.001**
**≤5**	**51 (22.7%)**	**69 (30.7%)**		
**>5**	**23 (10.2%)**	**82 (36.4%)**		
**Tumor number**			**1.494**	**0.222**
**Single**	**59 (26.2%)**	**109 (48.4%)**		
**Multiple**	**15 (6.7%)**	**42 (18.7%)**		
**Recurrence**			**6.347**	**0.012**
**No**	**48 (21.3%)**	**71 (31.6%)**		
**Yes**	**26 (11.6%)**	**80 (35.6%)**		
**Hepatitis**			**1.073**	0.300*
**No**	**4 (1.8%)**	**16 (7.1%)**		
**Yes**	**70 (31.1%)**	**135 (60.0%)**		
**Metastasis**			**6.863**	**0.009**
**No**	**63 (28.0%)**	**104 (46.2%)**		
**Yes**	**11 (4.9%)**	**47 (20.9%)**		

*Correction for continuity chi square test

### Prognostic values of the IHC signature

**Fig 5:**
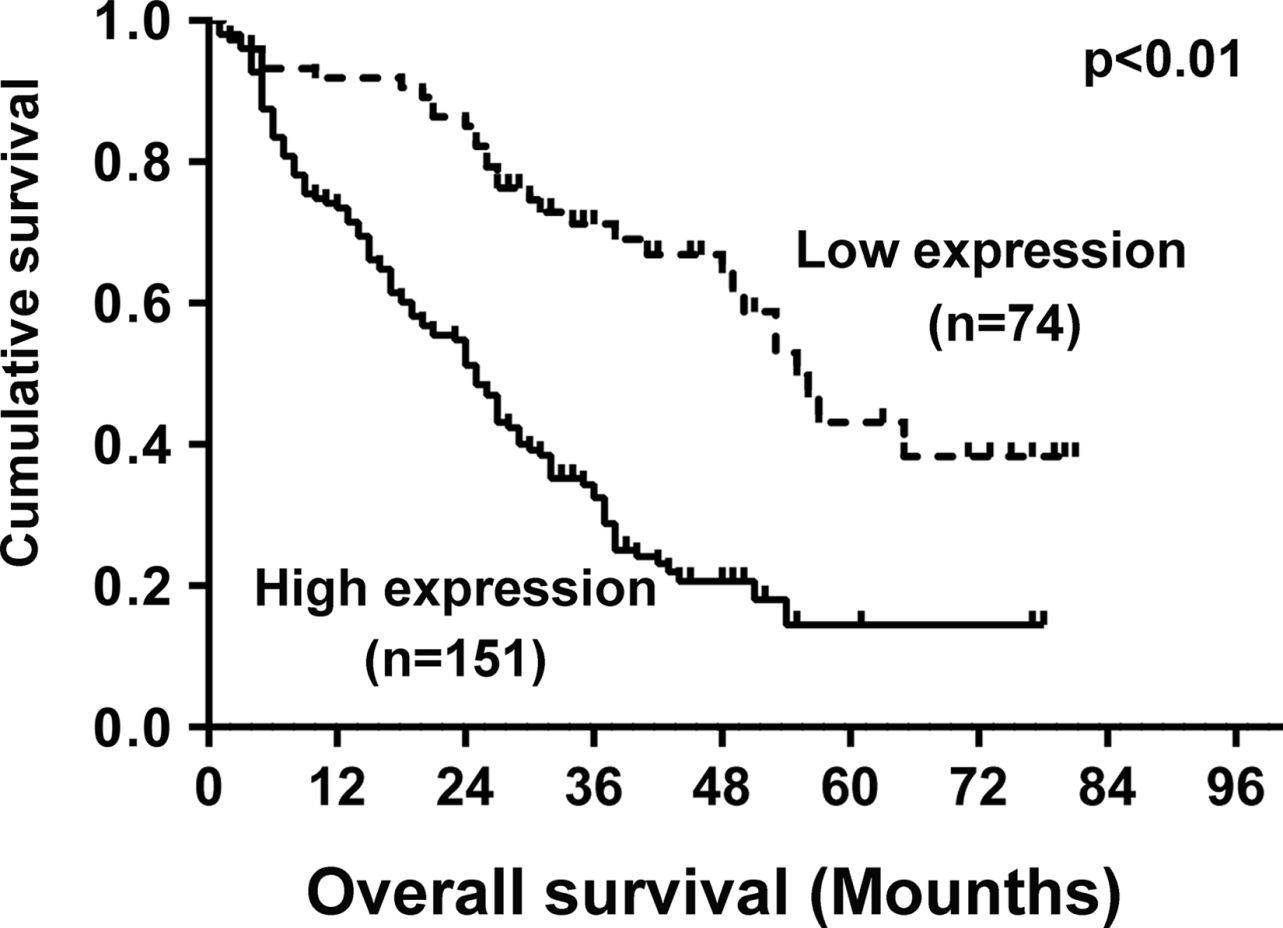
Kaplan–Meier survival analysis of the correlation between *SETD7* expression in patients with HCC and OS. Patients with high *SETD7* expression (solid line) exhibited decreased OS.

Kaplan–Meier analyses were performed to investigate the relationship between SETD7 expression and clinical prognosis. Patients with high SETD7 expression exhibited a significantly shorter overall survival (OS) than those with low SETD7 expression (Fig 5). The univariate analysis revealed obvious association of clinical parameters with OS. High SETD7 expression [95% confidence interval (*CI*), 2.026–4.607; p<0.001], AFP value of >200 ng/ml [95% *CI*, 1.004–1.956; p = 0.047], tumor size of >5cm [95% *CI*, 1.349–2.629; p<0.001], multiple tumors [95% *CI*, 1.085–2.225; p = 0.016], recurrence [95% *CI*, 1.277–2.495; p = 0.001], metastasis [95% *CI*, 1.522–3.074; p<0.001] and poor tumor differentiation [95% *CI*, 1.005–1.947; p = 0.047] are associated with reduced OS. Additionally, the multivariate analyses of cox proportional hazards model was used to analyze the relationship of the factors, including SETD7 expression and OS in HCC patients with surgical resection. High SETD7 expression [hazard ratio (HR), 2.497; 95% *CI*, 1.632–3.821; P<0.001], tumor size (>5 cm) (HR, 1.605; 95% *CI*, 1.141–2.258; P = 0.007), and metastasis (HR, 1.722; 95% *CI*, 1.187–2.496; P = 0.004) are significant independent prognostic factors for poor OS (Table 3).

**Table 3:** Univariate and multivariate analyses of factors associated with survival and recurrence.

	univariate		multivariate	
Clinical characters	HR(95%CI)	p value	HR(95%CI)	p value
**SETD7 high expression**	**3.055(2.026–4.607)**	**<0.001**	**2.497(1.632–3.821)**	**<0.001**
**Age (>55 years)**	**0.991(0.713–1.379)**	**0.991**		
**Gender (male)**	**1.484(0.948–2.321)**	**0.084**		
**Liver cirrhosis (Yes)**	**1.248(0.833–1.867)**	**0.283**		
**AFP (>200 ng/ml)**	**1.401(1.004–1.956)**	**0.047**	**1.164(0.822–1.647)**	**0.393**
**Tumor differentiation (III-IV)**	**1.399(1.005–1.947)**	**0.047**	**1.223(0.871–1.719)**	**0.245**
**Tumor size (>5cm)**	**1.883(1.349–2.629)**	**<0.001**	**1.605(1.141–2.258)**	**0.007**
**Tumor number (multiple)**	**1.554(1.085–2.225)**	**0.016**	**1.419(0.974–2.066)**	**0.068**
**Recurrence (yes)**	**1.785(1.277–2.495)**	**0.001**	**1.406(0.986–2.003)**	**0.060**
**Hepatitis (yes)**	**1.708(0.867–3.363)**	**0.122**		
**Metastasis (yes)**	**2.163(1.522–3.074)**	**<0.001**	**1.722(1.187–2.496)**	**0.004**

HR, hazard ratio; *CI*, confidence interval

## Discussion

In this study, SETD7 is more highly expressed in HCC tumor tissues than in ANLTs. To identify the biological significance of *SETD7*, *SETD7* was knockdown in SMMC-7721 cells by small interfering RNA, for SMMC-7721 cell line is basically high expression of SETD7. On the other hand, *SETD7* was overexpressed in HepG2 cells through transfecting overexpression plasmid, for HepG2 cell line is basically low expression of SETD7. Furthermore, SETD7 is significantly correlated with the expression of ZBTB20, CDKN2D, ZSCAN26, ACTG2, and CPA5. ZBTB20 (also known as DPZF, HOF, and ZNF288), which belongs to the BTB/POZ zinc finger family of proteins [22], represses forkhead box O1 (FoxO1) expression in lung cancer cells and promotes cell growth. Overexpression of ZBTB20 exhibits a significantly increases percentage of cells in the S phase and reduced the percentage of cells in the G1/G0 phase [23]. CDKN2D (also named p19^INK4d^) which belongs to the INK4 family of cyclin-dependent kinase inhibitors, can bind to CDK4/6; overexpression of CDKN2D leads to cell-cycle arrest in both G1 and G2 phases [24]. By inference, SETD7 promoting cell proliferation is a complex process of multiple factors involved in.

As the sequence-specific transcriptional repressor of alpha fetoprotein (AFP) [25], ZBTB20 knockout mice show some genes differentially involve in growth, metabolism, and detoxification in the liver [26]. Overexpression of ZBTB20 in HCC is strongly associated with recurrence, metastasis, and vein invasion and is also an independent prognostic factor for HCC [27]. In addition, overexpression of CDKN2D reverses the effect of miR-451, which contributes to esophageal carcinoma malignancy [28]. These results support that SETD7 regulating ZBTB20 and CDKN2D plays an important role in HCC.

ZSCAN26 also known as SREZBP, ZNF187, SRE-ZBP, is member of the C_2_H_2_ zinc finger family of proteins exemplified by transcription factor IIIA and the Drosophila Kriippel protein, shows specific SRE-binding activity, and its molecular function conclude metal ion binding, nucleic acid binding and transcription factor activity [29, 30]. ACTG2 links with E-cadherin via β-catenin. Recent studies identified several heterozygous missense variants in ACTG2 in megacystis-microcolon-intestinal hypoperistalsis patients had occurred de novo or were inherited [31–34]. The research of CPA5 is focus on mast cell diseases in zebrafish [35–37].

SETD7 regulation of the *ZBTB20* and *CDKN2D* expression may involve complex mechanisms, such as methylation of both histone and non-histone proteins. Although the mechanism underlying such regulation remains unknown in the present study, which is our research limitation, the biological function and clinical significance of SETD7 in HCC still provide useful information.

Univariate and multivariate analysis revealed that high *SETD7* expression, large tumor size and metastasis are associated with reduced OS. Previous studies showed that the size of tumors, recurrence and metastasis are important determinants of prognosis in HCC, small tumor diameter, without metastasis associated with relatively improved survival and prognosis [21].

Aberrations in H3K4 methylation occur in various cancers. Upregulation of MLL1 and SMYD3 increases H3K4 methylation, as well as induces breast and colorectal cancer, fibrosarcoma, and HCC [3]. However, there are some reports to the contrary, for example, high level of H3K4me2 is associated with increased survival for patients with large-cell or squamous-cell lung carcinoma [38], and low levels of H3K4me2 indicate poor survival in resectable pancreatic adenocarcinoma and metachronous liver metastasis of colorectal cancer [39]. We assume that H3K4me2 level in different tissue cells contribute differently to cancer progression. In the current study, H3K4me2 is more highly expressed in HCC tumor tissues than in ANLTs, and high H3K4me2 level in HCC tumor tissues is correlated with poor survival. Hence, H3K4me2 may be a dependent factor associated with SETD7, which is correlated with HCC development and progression.

## Conclusions

This study determined that SETD7 plays a critical role in HCC by regulating the cell cycle, and SETD7 can be an independent prognostic factor for the OS of patients with HCC. These findings can provide new insights into the biological and clinical significance of SETD7 in HCC.

## Supporting Information

S1 Fig *SETD7* cDNA was cloned between *Eco*RⅠ/ *Bam*H Ⅰ sites in PCMV direction. (DOCX)

S1 Table Summary of clinicopatholgic characteristics. (DOCX)

S2 Table Sequence of primers for qRT-PCR. (DOCX)

S3 Table Antibody used in western blot and IHC. (DOCX)

S4 Table Interference sequence used in transfection. (DOCX)
