# Supplementary material for: Increased Expression of SETD7 Promotes Cell Proliferation by Regulating Cell Cycle and Indicates Poor Prognosis in Hepatocellular Carcinoma
Source: PLoS One. 2016 May 16;11(5):e0154939. doi: 10.1371/journal.pone.0154939 (PMC4868314; doi:10.1371/journal.pone.0154939)
Supplement: S1 Fig — (DOCX) [file pone.0154939.s001.docx]

**S1 Fig. *SETD7* cDNA was cloned between *Eco*R Ⅰ/ *Bam*H Ⅰ sites in PCMV direction**

| Vector map | Carrier instructions |
| --- | --- |
| 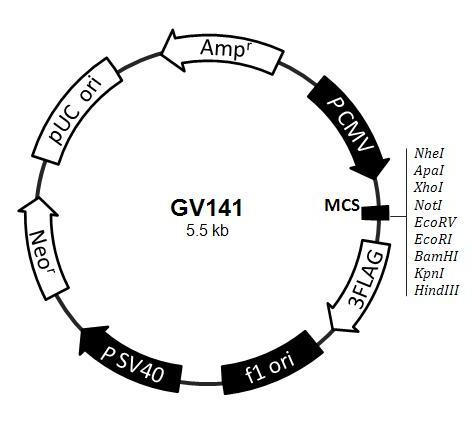 | Code of medium：GV141  Sequence element：CMV - MCS-3FLAG-SV40-Neomycin  Fluorescence labeling：no  Eukaryotic resistance：kana  Cloning site：*Eco*R Ⅰ/ *Bam*H Ⅰ  Insert name of gene：SETD7（NM_030648）  Source of inserted gene：Human |
