# Supplementary material for: Increased Expression of SETD7 Promotes Cell Proliferation by Regulating Cell Cycle and Indicates Poor Prognosis in Hepatocellular Carcinoma
Source: PLoS One. 2016 May 16;11(5):e0154939. doi: 10.1371/journal.pone.0154939 (PMC4868314; doi:10.1371/journal.pone.0154939)
Supplement: S1 Table — (DOCX) [file pone.0154939.s002.docx]

| **S1 Table. Summary of clinicopatholgic characteristics** | |
| --- | --- |
| Clinical character | No.of patients |
|  |  |
| SETD7 HCC |  |
| low expression | 74 |
| high expression | 151 |
| SETD7 ANLT |  |
| low expression | 183 |
| high expression | 42 |
| Age (y) |  |
| ≤55 | 116 |
| ＞50 | 109 |
| Gender |  |
| Male | 183 |
| Female | 42 |
| Liver cirrhosis |  |
| No | 55 |
| Yes | 170 |
| AFP (ng/ml) |  |
| ≤200 | 134 |
| ＞200 | 91 |
| Tumor differentiation |  |
| Ⅰ | 22 |
| Ⅱ | 96 |
| Ⅲ | 99 |
| Ⅳ | 8 |
| Tumor size (cm) |  |
| ≤5 | 120 |
| >5 | 105 |
| Tumor number |  |
| Single | 168 |
| Multiple | 57 |
| Recurrence |  |
| No | 119 |
| Yes | 106 |
| Hepatitis |  |
| No | 20 |
| Yes | 205 |
| Metastasis |  |
| No | 167 |
| Yes | 58 |
