# Supplementary material for: Increased Expression of SETD7 Promotes Cell Proliferation by Regulating Cell Cycle and Indicates Poor Prognosis in Hepatocellular Carcinoma
Source: PLoS One. 2016 May 16;11(5):e0154939. doi: 10.1371/journal.pone.0154939 (PMC4868314; doi:10.1371/journal.pone.0154939)
Supplement: S2 Table — (DOCX) [file pone.0154939.s003.docx]

**S2 Table. Sequence of primers for qRT-PCR**

| SETD7 | Forward (5’-3’) | CCTGGTGGAAGTTAGGTGCTA |
| --- | --- | --- |
|  | Reverse (5’-3’) | CGGTGTCTCTAATGCCTCTGA |
| ZBTB20 | Forward (5’-3’) | ATGCTAGAACGGAAGAAACCCA |
|  | Reverse (5’-3’) | TGTGAGCGTGAGAGTTTGTCA |
| CDKN2D | Forward (5’-3’) | TCACACTGCTGTGGTCAGCTTT |
|  | Reverse (5’-3’) | AGGATGTCCACGAGGTCCTGA |
| GAPDH | Forward (5’-3’) | TGCACCACCAACTGCTTAGC |
|  | Reverse (5’-3’) | GGCATGGACTGTGGTCATGAG |
