# Supplementary material for: Increased Expression of SETD7 Promotes Cell Proliferation by Regulating Cell Cycle and Indicates Poor Prognosis in Hepatocellular Carcinoma
Source: PLoS One. 2016 May 16;11(5):e0154939. doi: 10.1371/journal.pone.0154939 (PMC4868314; doi:10.1371/journal.pone.0154939)
Supplement: S3 Table — (DOCX) [file pone.0154939.s004.docx]

**S3 Table. Antibody used in western blot and IHC**

| Antibody | Brand | Catalog | Dilution | |
| --- | --- | --- | --- | --- |
|  |  |  | Western blot | IHC |
| GAPDH | Abcam | Ab8245 | 1:5000 |  |
| SETD7 | Abgent | AP1194c | 1:500 |  |
|  | Sigma | HPA058111 |  | 1:100 |
| H3K4me2 | Abcam | ab7766 | 1:1000 | 1:500 |
| CDKN2D | Abcam | ab102842 | 1:500 | 1:100 |
| ZBTB20 | Santa Cruz Biotechnology | sc-99728 | 1:500 | 1:75 |
| HRP-goat anti rabbit  HRP-Donkey anti mouse | Sango | D110058  D110085 | 1:10000 |  |
| EnVision Detection Systems Peroxidase/DAB, Rabbit/Mouse | DOKO | K5007 |  | 1:1 |
