# Supplementary material for: Increased Expression of SETD7 Promotes Cell Proliferation by Regulating Cell Cycle and Indicates Poor Prognosis in Hepatocellular Carcinoma
Source: PLoS One. 2016 May 16;11(5):e0154939. doi: 10.1371/journal.pone.0154939 (PMC4868314; doi:10.1371/journal.pone.0154939)
Supplement: S4 Table — (DOCX) [file pone.0154939.s005.docx]

**S4 Table. Interference sequence used in transfection**

| si-SETD7-1 | Target sequence | GGGCACCTGGATGACGGA |
| --- | --- | --- |
|  | Forward (5’-3’) | GGGCACCUGGACGAUGACGGA dTdT |
|  | Reverse (3’-5’) | dTdTCCCGUGGACCUGCUACUGCCU |
| si-SETD7-2 | Target sequence | GGAGTGTGCTGGATATATT |
|  | Forward (5’-3’) | GGAGUGUGCUGGAUAUAUU dTdT |
|  | Reverse (3’-5’) | dTdT CCUCACACGACCUAUAUAA |
| si-SETD7-3 | Target sequence | CAAACTGCATCTACGATAT |
|  | Forward (5’-3’) | CAAACUGCAUCUACGAUAU dTdT |
|  | Reverse (3’-5’) | dTdT CCUCACACGACCUAUAUAA |
